# Predicting survival post-cardiac arrest: An observational cohort study

**DOI:** 10.1016/j.resplu.2023.100447

**Published:** 2023-08-18

**Authors:** Ian R Drennan, Kevin E Thorpe, Damon Scales, Sheldon Cheskes, Muhammad Mamdani, Laurie J Morrison, Rescu Epistry Investigators

**Affiliations:** a Department of Emergency Services, Sunnybrook Health Science Centre, Toronto, ON, Canada; b Institute of Health Policy, Management and Evaluation, Dalla Lana School of Public Health, University of Toronto, Toronto, ON, Canada; c Division of Emergency Medicine, Department of Family and Community Medicine, Temerty Faculty of Medicine, University of Toronto, Toronto, ON, Canada; d Applied Health Research Centre, St. Michael’s Hospital, Toronto, ON, Canada; e Department of Critical Care, Sunnybrook Health Sciences Centre, Toronto, ON, Canada; f Sunnybrook Centre for Prehospital Medicine, Sunnybrook Health Sciences Centre, Toronto, ON, Canada; g Data Science and Advanced Analytics, St. Michael’s Hospital, Toronto, ON, Canada; h Rescu, Li Ka Shing Knowledge Institute, Department of Emergency Medicine, St. Michael’s Hospital, Toronto, ON, Canada; i Division of Emergency Medicine, Department of Medicine, Temerty Faculty of Medicine, University of Toronto, ON, Canada

**Keywords:** Post-Cardiac Arrest, Out-of-Hospital Cardiac Arrest, Prediction, Resuscitation

## Abstract

**Introduction:**

Over 400,000 out-of-hospital cardiac arrest (OHCA) occur each year in Canada and the United States with less than 10% survival to hospital discharge. Cardiac arrest is a heterogenous condition and patient outcomes are impacted by a multitude of factors. Prognostication is recommended at 72 hours after return of spontaneous circulation (ROSC), however there may be other factors that could predict patient outcome earlier in the post-arrest period. The objective of our study was to develop and internally validate a novel clinical prediction rule to risk stratify patients early in the post-cardiac arrest period.

**Methods:**

We performed a retrospective cohort study of adult (≥18 years) post-cardiac arrest patients between 2010 and 2015 from the Epistry Cardiac Arrest database in Toronto. Our primary analysis used ordinal logistic regression to examine neurologic outcome at discharge using the modified Rankin Scale (mRS). Our secondary analysis used logistic regression for neurologic outcome and survival to hospital discharge. Models were internally validated using bootstrap validation.

**Results:**

A total of 3432 patients met our inclusion criteria. Our clinical prediction model was able to predict neurologic outcome on an ordinal scale using our predefined variables with an AUC of 0.89 after internal validation. The predictive performance was maintained when examining neurologic outcome as a binary variable and survival to hospital discharge.

**Conclusion:**

We were able to develop a model to accurately risk stratify adult cardiac arrest patients early in the post-cardiac arrest period. Future steps are needed to externally validate this model in other healthcare settings.

## Introduction

Despite improvements in resuscitation science and treatment, survival rates from out-of-hospital cardiac arrest (OHCA) remain low.^1^ Two-thirds of patients who have a return of spontaneous circulation (ROSC) and are admitted to hospital will die, most as a result of neurologic injury.^2^ Guidelines recommend waiting at least 72 hours post-ROSC to make prognostication decisions.^3^ This time interval allows for recovery from ischemic-reperfusion injury, clearance of medication such as sedatives and paralytics, and recovery after targeted temperature management.^4^, ^5^, ^6^ This uncertainty during the early post-cardiac arrest period makes discussions regarding goals of care and therapeutic planning difficult with patient families. Risk stratification of patients can help to reduce uncertainty during this period and inform discussions around patient care. In addition, risk stratification can have many important roles for patient identification in future research studies and in quality improvement initiatives to help benchmark within and across institutions.

Many patient characteristics, intra-arrest and early post-arrest variables are associated with patient outcomes. For example, the Utstein data elements (age, sex, location of arrest, witness status, bystander CPR, initial cardiac rhythm, EMS response time) have been shown to predict 72% of the variability in patient outcomes.^7^

Other prediction models have been developed which have shown good to excellent predictive performance in the derivation population, however most fail to demonstrate reliability during validation for use in practice.^8^, ^9^, ^10^, ^11^

Our goal was to derive and internally validate a clinical prediction model for use early (24 hours) after return of spontaneous circulation to predict survival and good functional outcome at hospital discharge.

## Methods

### Study design and setting

We performed a retrospective cohort study using data from the Toronto Regional RescuNET Epistry Cardiac Arrest Database (Rescu Epistry). Rescu Epistry is compliant with the Resuscitation Outcomes Consortium (ROC) Epistry-Cardiac Arrest and based on the Strategies for Post Arrest Resuscitation Care (SPARC) methodologies described elsewhere.^12^, ^13^ Rescu Epistry is a prospective population-based registry of consecutive OHCAs that occur within 7 geographical regions in southern Ontario. Toronto Regional RescuNET has a population of 6.6 million people and is served by 7 land paramedic agencies (Toronto, Peel, York, Halton, Durham, Simcoe, and Muskoka), a provincial air ambulance service (Ornge) and 42 destination hospitals. This protocol was approved by the lead hospital Research Ethics Board (REB) at St. Michael’s Hospital.

### Population

We included consecutive adult (≥18 years) OHCA treated by paramedics that occurred between January 1, 2010 and December 31, 2015 and had a documented ROSC in hospital for greater than 20 minutes. We excluded OHCA that were not treated by paramedics due to criteria for obvious irreversible death (e.g. rigor mortis, lividity), patients with cardiac arrests of obvious non-cardiac etiology (e.g. trauma), patients who had a valid “do not resuscitate” (DNR) order in the prehospital setting or within the first 2 hours after ROSC in the hospital, and patients who died within the first 24-hours after ROSC as they were not eligible to be assessed for some of our variables of interest.

### Outcomes and included variables

Variables of interest (age, sex, initial cardiac rhythm, location, bystander CPR, witness status, EMS response time interval, etiology of cardiac arrest) were all consistent with Utstein definitions.^14^, ^15^ EMS response time interval was defined as the time from call to 911 dispatch to arrival on scene of the first 911-initiated first responder (paramedics or fire department personnel). Duration of resuscitation was defined as the time from EMS initiation of CPR to the time of first ROSC (prehospital or in-hospital), or termination of resuscitation in those patients without a documented ROSC. In cases where the timing of ROSC was not recorded any patient who obtained ROSC in the prehospital setting the timing of ROSC was imputed as the time of arrival at the ED. For patients without ROSC in either the prehospital or in-hospital setting where the timing of termination of resuscitation was not recorded the resuscitation was assumed to stop 30 minutes after arrival at the ED. Blood gas values (pO2, pCO2, HCO3- were defined as the lowest obtained value within the first 24 hours. If multiple pH values were obtained we used the first obtained value. GCS values were defined as the highest total score and highest score for the motor component recorded over the first 24 hours post-ROSC.

The primary outcome was functional survival at hospital discharge. Functional survival was evaluated using the modified Rankin Scale (mRS)^16^ and defined as a good functional outcome (0–2), poor functional outcome (3–5) and dead (6). Our secondary outcomes were survival to hospital discharge and functional survival as a binary outcome [good (mRS 0–2) and poor (mRS 3–6)].^16^

### Analysis

Our goal was to derive and internally validate a clinical prediction model for early post-cardiac arrest patients. We used descriptive bivariate analyses (ANOVA [or Student’s t-test] and Pearson’s chi-squared test) to examine the association of predetermined variables of interest and patient outcomes.

#### Model derivation

For our primary analysis we derived our early predictive model using ordinal logistic regression. Our outcome for this model was mRS score at hospital discharge categorized as “good” (mRS 0–2), “poor” (mRS 3–5) and “dead” (mRS 6). To avoid inherent biases generated from using stepwise variable reduction^17^ we included predetermined variables of interest based on a review of the literature and expertise: age (continuous), EMS response time (continuous), sex (male/female), location (public/private), witness status (not witnessed, bystander, EMS), bystander CPR (yes/no), and initial cardiac rhythm (ventricular fibrillation/ventricular tachycardia vs. pulseless electrical activity/asystole), duration of resuscitation, pupillary response to light during the first 24-hours post-cardiac arrest, maximum Glasgow coma scale (GCS) motor score in the first 24-hours post-cardiac arrest. We examined use of corneal reflexes, however there was a large proportion of missing values and so this variable was removed from the analysis. In addition, we derived a second early prediction model that included arterial blood gas values during the first 24-hours post-cardiac arrest (pO2, pCO2, pH, and bicarbonate).

For our secondary analysis, we derived early prediction models examining functional status as a binary variable, with good outcome defined as mRS 0–2 and poor outcome mRS 3–6, and for survival status at hospital discharge (Alive/Dead). The results of our regression models are presented with odds ratios (OR) and corresponding 95% confidence intervals. The odds ratio for an ordinal regression model is the odds of moving from one level to the next within each variable. Our models met the assumptions for proportional odds ordinal regression and therefore we assumed that the odds of moving across each level was proportional within variables. Continuous variables were maintained in their original form and we used restricted cubic splines to examine for non-linearity. We performed a likelihood ratio test, as a global test for additivity, to compare models with and without non-linear and interaction terms as well as to compare models with additional arterial blood gas variables. The significance of the inclusion of these additional terms was represented with a *P* value <0.30.

#### Internal validation

Internal validation of our final model was performed using Bootstrap 0.632 validation.^18^ We performed 100 bootstrap repetitions and model performance statistics were averaged across the samples and subtracted from the original performance metrics to determine the “optimism-corrected” performance metrics for the model.

#### Model performance

We assessed model performance using Brier statistic as an overall measure of performance and specific measures of model discrimination and calibration.^19^ Discrimination (ability of model to differentiate between those with and without the outcome) was assessed using c-statistic and area under the receiver operating characteristic (AUC/ROC) curve. Calibration (agreement between predicted and observed outcomes) was evaluated with the use of calibration curves as well as calibration slope and intercept. Our final model is presented as a nomogram.

All statistical computing was done using R: A language and environment for statistical computing (Vienna, Austria).

## Results

**Fig. 1:**
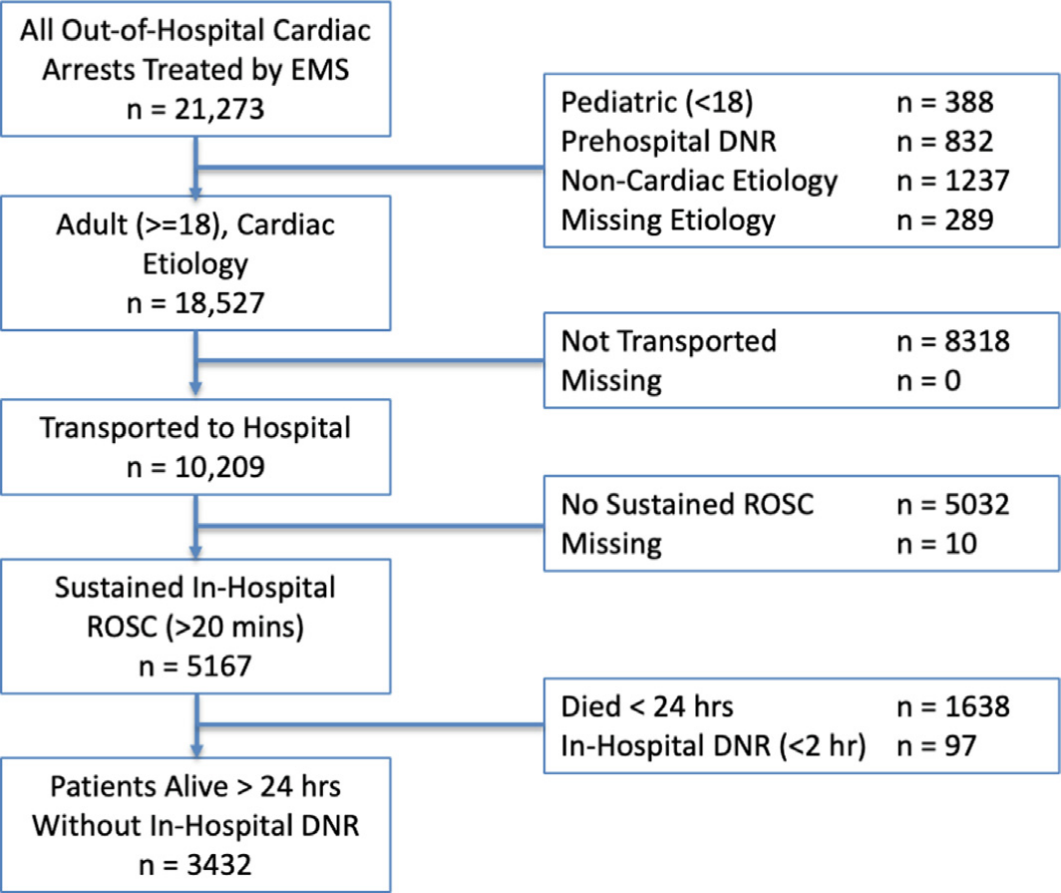
Patient flow diagram.

Overall 5167 OHCA patients had a return of spontaneous circulation during our study time period, of which 3432 met our inclusion criteria. (Fig. 1) Of the included patients, 1656 (48%) survived to hospital discharge and 1452 (42%) had a good functional outcome (mRS 0–2). Seventy-seven patients were missing functional outcome and were removed from further analysis.

The median age of patients treated by paramedics was 65.6 years (IQR 54.5, 77.1) and 70% of patients were male. The descriptive statistics of included patients based on functional status at hospital discharge are shown in Table 1. There was a significant difference found based on functional status at discharge (good functional status vs poor neurological status vs dead) in terms of all of our pre-determined variables of interest.

**Table 1:** Comparison of post-cardiac arrest patients by functional outcome at hospital discharge (*n* = 3355).

Variable		mRS 0–2	mRS 3–5	mRS 6
		*n* = 1452	*n* = 145	*n* = 1758
18–39		61.0 (52.1, 70.9)	62.6 (52.3, 70.9)	70.2 (58.5, 80.3)
40–59		109 (8)	12 (8)	86 (5)
60–74		535 (37)	46 (32)	374 (21)
75+		272 (19)	37 (26)	724 (41)
EMS Response Time (min), med (IQR)		5.8 (4.7, 7.1)	5.7 (4.6, 7.0)	6.0 (5.0, 7.3)
<4		215 (16)	20 (16)	172 (11)
4–10		989 (76)	96 (77)	1327 (83)
4+		101 (8)	9 (7)	105 (7)
Cardiac Arrest Duration (min), med (IQR)		13.0 (7.0, 20.0)	18.1 (12.8, 24.0)	23.3 (17.2, 31.0)
Duration of Resus (min), med (IQR)		6.3 (3.0, 13.0)	11.0 (7.9, 24.0)	16.0 (11.0, 24.0)
Male Sex		1094 (75)	99 (68)	1157 (66)
Female Sex		358 (25)	46 (32)	601 (34)
Public Location		581 (40)	46 (32)	404 (23)
Private Location		871 (60)	99 (68)	1354 (77)
Initial Shockable Rhythm		1087 (75)	78 (54)	602 (34)
Initial Non-Shockable Rhythm		359 (25)	66 (46)	1155 (66)
Patient Comorbidities				
Cardiac (Afib, MI, CHF)		679 (47)	64 (44)	890 (51)
Hypertension		780 (54)	76 (52)	1052 (60)
Diabetes		307 (21)	38 (26)	605 (34)
Respiratory		255 (18)	26 (18)	488 (28)
Cancer		114 (8)	12 (8)	215 (12)
Prehospital Advanced Life Support Care		1341 (92)	139 (96)	1679 (96)
Intravenous/Intraosseous Attempted		1310 (91)	136 (95)	1646 (94)
Epinephrine Administration		439 (31)	92 (65)	1400 (80)
Advanced Airway (ETT or SGA)		610 (43)	104 (74)	1476 (85)
Witness Status				
EMS Witnessed		426 (30)	27 (19)	301 (17)
Bystander Witnessed		826 (57)	83 (57)	889 (51)
Unwitnessed		191 (13)	35 (24)	559 (32)
CPR Status				
EMS CPR		426 (29)	27 (19)	301 (17)
Bystander CPR		653 (45)	53 (37)	686 (39)
No Bystander CPR		366 (25)	64 (44)	759 (43)
Bystander AED Application		153 (23)	10 (18)	52 (7)
Pupillary Response at 24 hrs		1021 (93)	110 (82)	867 (55)
Corneal Reflexes at 24 hrs		118 (81)	8 (40)	70 (23)
Max GCS Score at 24 hrs		7.0 (3.0, 15.0)	3.0 (3.0, 6.0)	3.0 (3.0, 4.0)
Max GCS Motor Score at 24 hrs		4.0 (1.0, 6.0)	1.0 (1.0, 4.0)	1.0 (1.0, 1.0)
Minimum pH level of 24 hrs		7.3 (7.2, 7.3)	7.2 (7.1, 7.3)	7.2 (7.0, 7.3)
Initial pO2 post-ROSC		121 (86, 197)	103 (78, 157)	128 (85, 224)
Initial pCO2 post-ROSC		43 (37, 50)	42 (36, 50)	45 (37, 60)
Initial Bicarbonate post-ROSC		20 (17, 22)	19 (16, 22)	18 (15, 21)

*77 patients removed for missing functional outcome. mRS = modified rankin scale; Yr = year; IQR = interquartile range; med = median; EMS = emergency medical service; min = minute; ETT = endotracheal tube; SGA = supraglottic airway; CPR = cardiopulmonary resuscitation; MI = myocardial infarction; CHF = congestive heart failure; afib = atrial fibrillation; AED = automated external defibrillator; hrs = hours; pO2 = arterial partial pressure of oxygen; pCO2 = aterial partial pressure of carbon dioxide; GCS = Glasgow coma scale; ROSC = return of spontaneous circulation.

**Fig. 2:**
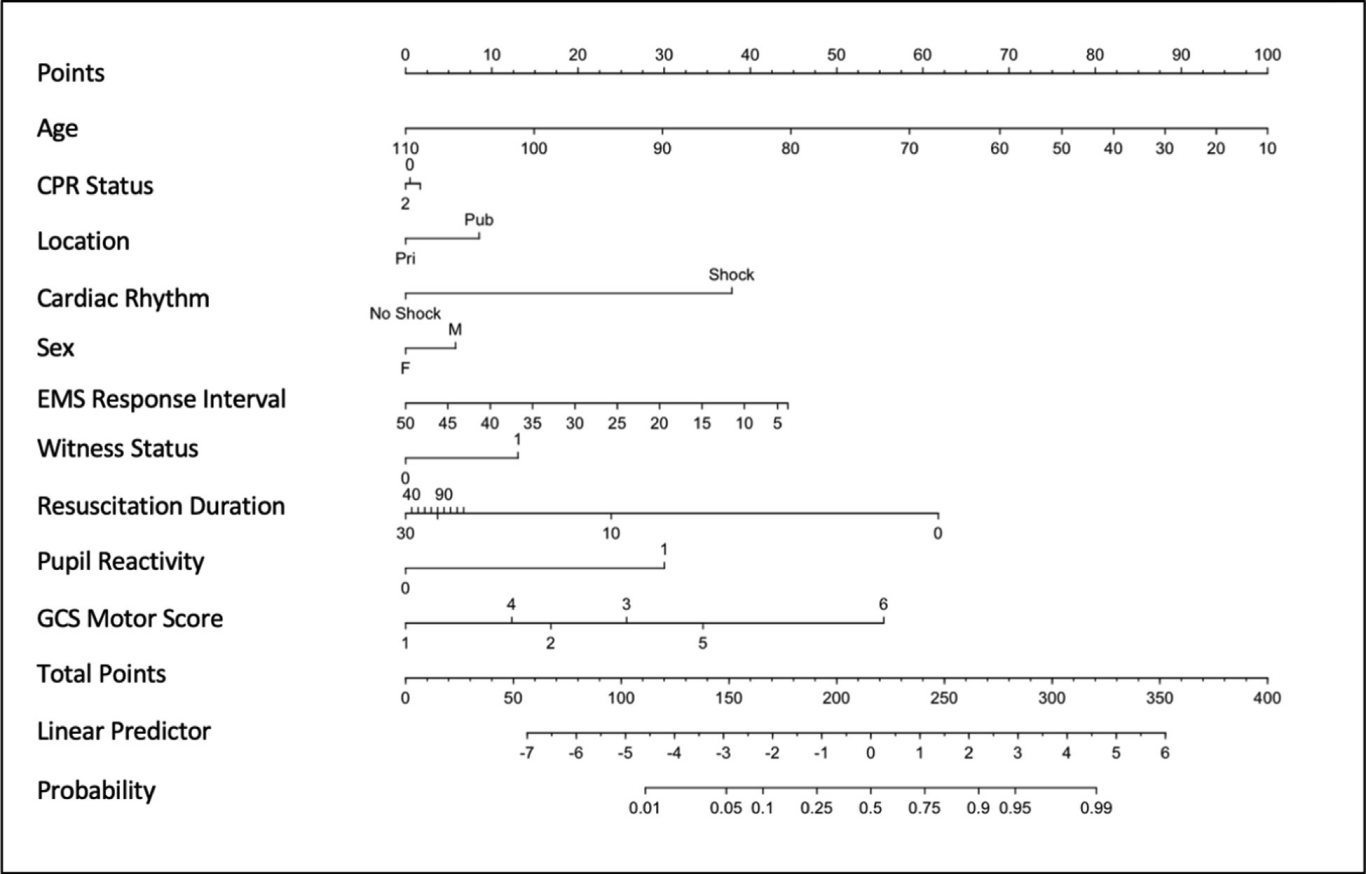
Nomogram of final model. Note: CPR Status 0 = no bystander CPR; 1 = bystander CPR; 2 = EMS Witnessed/CPR. Witness Status 0 = unwitnessed; 1 = witnessed Age and Resuscitation Duration are both nonlinear terms The nomogram is used by drawing a line from a given value in each predictor to the points line. The sum of all the points is then mapped to the Total Points axis. The linear predictor and the probability of survival can than be obtained from the corresponding axis.

**Fig. 3:**
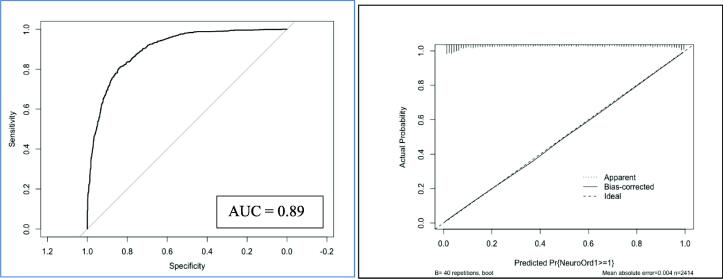
Model performance for functional outcome at hospital discharge using ordinal regression.

The results of our predictive model are presented in Fig. 2 and Table 2. Our primary model was found to have high predictive ability for functional outcome at hospital discharge. The overall predictive performance, measured with Brier statistic, was 0.127. The discrimination of the model was excellent with a c-statistic (AUC/ROC) of 0.890 (Table 3 and Fig. 3) and the calibration plot showed excellent calibration across the range of predicted probabilities. (Fig. 3) Our model performance statistics were maintained on internal validation suggesting very little overfitting in our model (Table 3). EMS response time and duration of resuscitation were kept as continuous variables and non-linear terms were found to improve the predictive performance of our model (LR test *P* < 0.001). The addition of blood gas values (pCO2, pO2, pH, HCO3-) did not increase the predictive performance of our model, however increased the model complexity and were subsequently removed (Appendix A).

**Table 2:** Ordinal regression analysis for determining functional outcome in post-cardiac arrest patients (*n* = 2414).

Variable	Odds Ratio	95% CI	*P* value
Age 18–60 (/yr)	0.97	0.96, 0.99	<0.001
Age 70–103 (/yr)	0.93	0.92, 095	<0.001
EMS Response Time 1–5 (/min)	0.96	0.86, 1.08	0.167
EMS Response Time 6–24 (/min)	0.96	0.90, 1.02	0.167
Male Sex (Ref Female)	1.19	0.93, 1.53	0.168
Private Location (Ref Public)	1.43	1.12, 1.81	0.003
Initial Shockable Rhythm (Ref Non-Shockable)	4.57	3.57, 5.86	<0.001
Witnessed* (Ref Unwitnessed)	1.77	1.34, 2.34	<0.001
EMS CPR (Ref None)	1.01	0.71, 1.44	0.999
Bystander CPR (Ref None)	1.00	0.78, 1.28	0.999
Presence of Pupillary Reflexes (Ref Absent)	3.57	2.67, 4.77	<0.001
Max Motor GCS Score 6 (Ref = 1)	10.67	7.41, 15.36	<0.001
Max Motor GCS Score 5 (Ref = 1)	4.30	2.61, 7.09	<0.001
Max Motor GCS Score 4 (Ref = 1)	1.79	1.18, 2.71	<0.001
Max Motor GCS Score 3 (Ref = 1)	2.79	1.75, 4.44	<0.001
Max Motor GCS Score 2 (Ref = 1)	1.90	1.11, 3.23	<0.001
Duration of Resuscitation 1–17 (/min)	0.87	0.85, 0.89	<0.001
Duration of Resuscitation 25–90 (/min)	1.00	0.99, 1.02	<0.001

*includes EMS and bystander witnessed. Yr = year; EMS = emergency medical services; min = minute; CPR = cardiopulmonary resuscitation; CI = confidence interval; Ref = reference; GCS = Glasgow Coma Scale.

LRTEST < 0.001 for inclusion of non-linear terms (Age, EMS Response, Duration of Resuscitation).

P Values for Age (8.87e-32), Initial Rhythm (6.19e-34), Witnessed (2.80e-05), Pupillary Reflexes (1.89e-17), GCS Motor Score (7.74e-40), and Duration of Resuscitation (4.03e-38).

**Table 3:** Summary performance statistics for original ordinal regression model and internal validation.

**Model Summary Statistics**	**Model**	**Optimism**	**Bootstrap Validation**
AIC	2565.9	n/a	n/a
R2	0.563	0.009	0.555
Somer’s Dxy	0.780	0.007	0.772
Slope	1.000	0.029	0.971
Brier Statistic	0.127	−0.003	0.129
c-statistic	0.890	0.004	0.886
% of outcome explained by model	78.0%	0.80%	77.2%

As a secondary analysis both models had a similar performance as the primary analysis for the outcomes of functional status at hospital discharge as a binary variable and for survival to hospital discharge (Appendices B and C).

The primary analysis was repeated after performing multiple imputation for missing data. There were only very minor differences noted between the imputed model and the original complete case model supporting the robustness of our analysis (Appendix D).

## Discussion

In this analysis of prospectively collected, consecutive post-cardiac arrest patients we derived and internally validated an early clinical model to predict functional outcome at hospital discharge with excellent discrimination and calibration. The model performance was maintained when functional outcome was examined as a binary variable, for survival to hospital discharge, and with imputation for missing data.

There are a number of other prediction models that have been derived in the cardiac arrest literature.^20^, ^21^ Few of the prediction models, however, have undergone rigorous external validation, and those that have suffer in their performance on external data.^20^ Our model development has some strengths compared to previous research. In order to be applied in clinical practice, clinical prediction rules must undergo rigorous methodological development. In this study we completed the initial derivation and internal validation of our prediction model on a large, research-quality prospective registry with pre-specification of model parameters using previous literature and clinical expertise. Our derivation was completed avoiding the use of traditional stepwise variable selection strategies which can result in overly optimistic models that perform poorly on external validation.^17^ We also avoided the use of treatment variables which can be subjective and vary over time with changes in practice, making prediction models obsolete. Pupillary response is one variable that may change over time, as the ability to assess pupillary response during the first 24 hours becomes easier as treatment moves from temperature management to fever control. More accurate assessment of pupillary response we feel may improve the performance of our model as this has been shown to have high prognostic performance post re-warming after TTM.^4^, ^5^ Our model maintained its performance accuracy on internal validation as well demonstrating the robustness of our model and the potential for high predictive performance on future external validation.

A properly derived and validated clinical prediction rule may serve many purposes in resuscitation science. Risk stratification of patients in future cardiac arrest research may help to identify groups of patients who are more likely to benefit from specific interventions. Cardiac arrest patients are a heterogenous group where characteristics of the event and of the patient are associated with patient outcomes and poor overall survival. Further, ischemic injury during cardiac arrest depends on a number of factors such as underlying health status, time until treatment, duration of cardiac arrest. Within the cardiac arrest population there are patients with extremely low probability of survival (such as those with asystole initial cardiac rhythm) in which treatment may not be beneficial. There is also a subset of patients (such as those with very short down time) in which the probability of a good outcome is high regardless of the treatment provided. There may be a third subset of patients which may have the largest degree of benefit from treatment. Enrolment of diverse cardiac arrest patients in clinical trials in which the majority of patients die may washout the effect of an intervention. As a point in fact, there are a number of large, randomized trials from North America and Europe with a neutral outcome over the past decade.^22^, ^23^, ^24^, ^25^ A validated clinical prediction rule could be reliably applied by trialists to identify the patients who are more likely to benefit from the treatment. This would refine the trials at the point of enrolment based on their clinical prediction to have a favourable outcome. This approach may enable trialists to identify treatments that can improve outcomes for specific groups of cardiac arrest patients and perhaps contribute to reducing the number of neutral trials in resuscitation.

Second, risk stratification models can be used to compare risk-adjusted mortality within, and across institutions for quality improvement, benchmarking, and reporting. Risk-adjusted outcomes could help to advance the science behind specialized cardiac arrest centres for post-cardiac arrest care. Further, it could be used for reporting purposes to compare across institutions for things such as quality improvement initiatives and funding for specialized centres, similar to how TIMI risk scores is used for risk-adjustment of cath labs for ST-segment myocardial infarction (STEMI) patients.^26^

Third, risk stratification models could provide some direction at the bedside during the early post-ROSC period. While there are currently no prediction models that have consistently shown high enough accuracy to be used at the bedside for post-ROSC patients a highly accurate validated clinical prediction rule would provide guidance during the early post-cardiac arrest period to have conversations with families on patient prognosis. The gender bias towards withdrawing care early or choosing a less invasive approach in women post arrest could be addressed by a validated early clinical prediction rule.^27^ It would be easier to guide discussions with the family based on a predicted favourable outcome. It could also be used to help guide decisions on treatment that may be useful for subsets of post-cardiac arrest patients, or to inform decisions on the continuation of post-arrest management. In addition, something that has gained attention during the COVID-19 pandemic is the allocation of scarce resources. One difficulty during these times is determining how to use a finite number of resources for a large influx of ICU and critically ill patients. In order to properly triage resources during these extreme times it is critical to accurately determine the probability of a patient’s outcome to allow for appropriate resource utilization for the greatest number of patients.

### Limitations

There are some important limitations with our study. It is a retrospective cohort study and is limited by the use of previously collected data. As this is an observational design we are unable to determine cause and effect relationships between our variables and outcomes of interest. We had a high number of missing values in some key variables of interest such as corneal reflexes and were unable to examine these variables in our model. While the model had excellent predictive performance there are still unknown variables that could improve the overall performance. Lastly, our model requires external validation prior to use.

## Conclusion

Using a novel combination of intra-arrest and early post-cardiac arrest variables we were able to derive and internally validate a prediction model that had excellent predictive accuracy for predicting functional outcome at hospital discharge.

## Funding

The Resuscitation Outcomes Consortium Epistry study is supported by a cooperative agreement (5U01 HL077863) with the National Heart, Lung, and Blood Institute in partnership with the National Institute of Neurological Disorders and Stroke, Canadian Institutes of Health Research–Institute of Circulatory and Respiratory Health, Defense Research and Development Canada, Heart and Stroke Foundation of Canada, and American Heart Association. Rescu Epistry is funded by a centre grant from the Laerdal Foundation, and knowledge translation collaborative grants and operating grants from Canadian Institutes of Health Research and the Heart and Stroke Foundation of Canada.

IRD was funded by the Canadian Institute of Health Research (CIHR) Banting and Best Doctoral Research Award.

## Declaration of Competing Interest

The authors declare that they have no known competing financial interests or personal relationships that could have appeared to influence the work reported in this paper.

## Figures and Tables

**Table:** 

Barto Nascimiento	barto.nascimento@sunnybrook.ca
Damon Scales	damon.scales@sunnybrook.ca
Dennis Ko	dennis.ko@ices.on.ca
Jamie Hutchison	jamie.hutchison@sickkids.ca
Katie Dainty	daintyk@smh.ca
Laurie Morrison	morrisonl@smh.ca
Paul Dorian	dorianp@smh.ca
Richard Swartz	rick.swartz@sunnybrook.ca
Richard Verbeek	rverbeek@socpc.ca
Sandro Rizoli	rizolis@smh.ca
Sheldon Cheskes	scheskes@socpc.ca
Steven Brooks	brookss1@kgh.kari.net
Steve Lin	lins@smh.ca
Tim Chan	tcychan@mie.utoronto.ca

## Appendix A

RESCU Epistry Investigators
