# Supplementary material for: Predicting survival post-cardiac arrest: An observational cohort study
Source: Resusc Plus. 2023 Aug 18;15:100447. doi: 10.1016/j.resplu.2023.100447 (PMC10470201; doi:10.1016/j.resplu.2023.100447)

APPENDICES / SUPPLEMENTAL RESULTS

A: Results of Ordinal Regression Analysis including blood gas values

Table A-1: Ordinal regression analysis for functional outcome at hospital discharge

CI = confidence interval; yr = year; EMS = emergency medical services; min = minute; ref = reference; CPR = cardiopulmonary resuscitation; GCS = Glasgow coma scale;

Table A-2: Model performance statistics for original model and internal validation

Figure A-1: Calibration curve for ordinal regression model


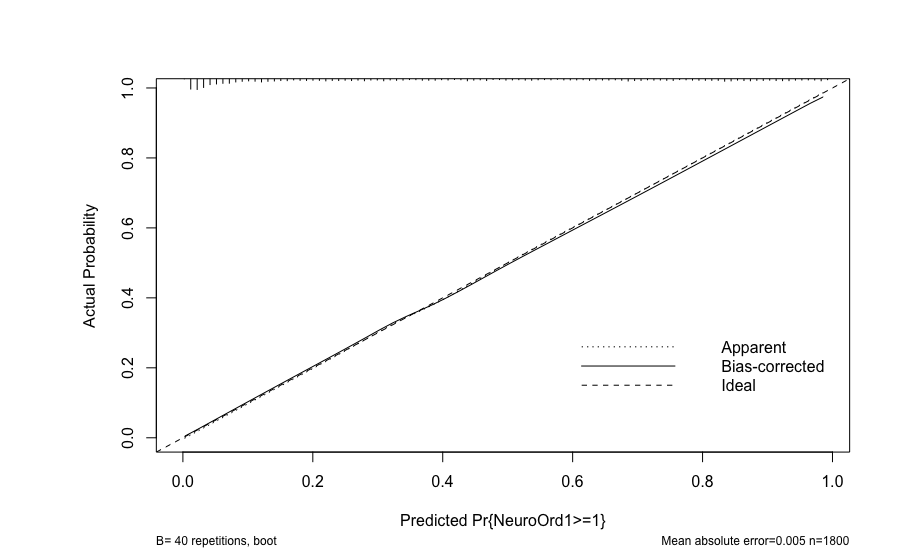


B: Results of logistic regression analysis for functional outcome

Table B-1: Multivariable regression analysis for functional outcome at hospital discharge

CI = confidence interval; yr = year; min = minute; ref = reference; EMS = emergency medical services; CPR = cardiopulmonary resuscitation; GCS = Glasgow coma scale

Table B-2: Performance statistics for original model and internal validation

Figure B-1: Calibration curve for logistic regression model


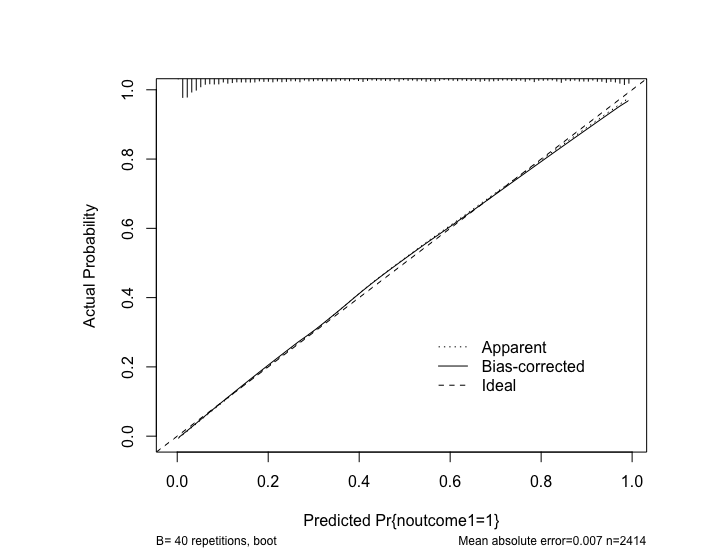


C: Results of logistic regression analysis for survival

Table C-1: Multivariable logistic regression model for survival to hospital discharge

CI = confidence interval; Yr = year; min = minute; EMS = emergency medical services; CPR = cardiopulmonary resuscitation; ref = reference; GCS = Glasgow coma scale; Max = maximum

Table C-2: Model performance statistics for original model and internal validation

Figure C-1: Calibration curve for logistic regression model


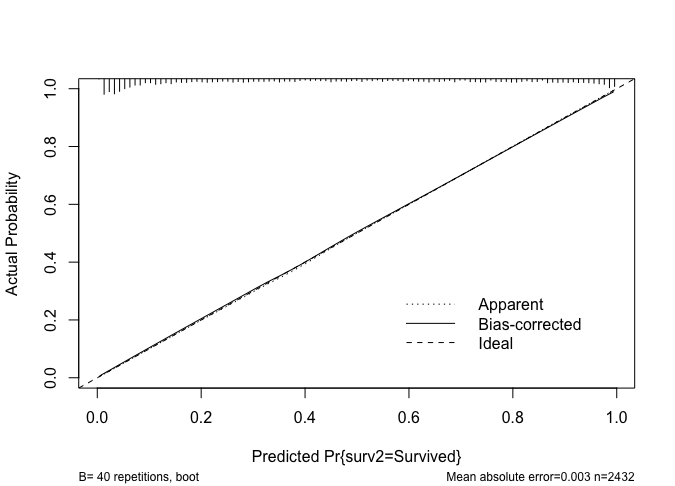


D: Results of ordinal regression analysis with multiple imputation

Table D-1: Ordinal Regression Model for functional outcome at hospital discharge with multiple imputation

*includes bystander and EMS witnessed; Nonlinear effect for duration of resuscitation, age, EMS response (LRTEST < 0.001); CI = confidence interval; Yr = year; EMS = emergency medical services; min = minute; ref = reference; CPR = cardiopulmonary resuscitation; Max = maximum; hrs = hours; GCS = Glasgow coma scale

Appendix E. Formula for Fitted Model


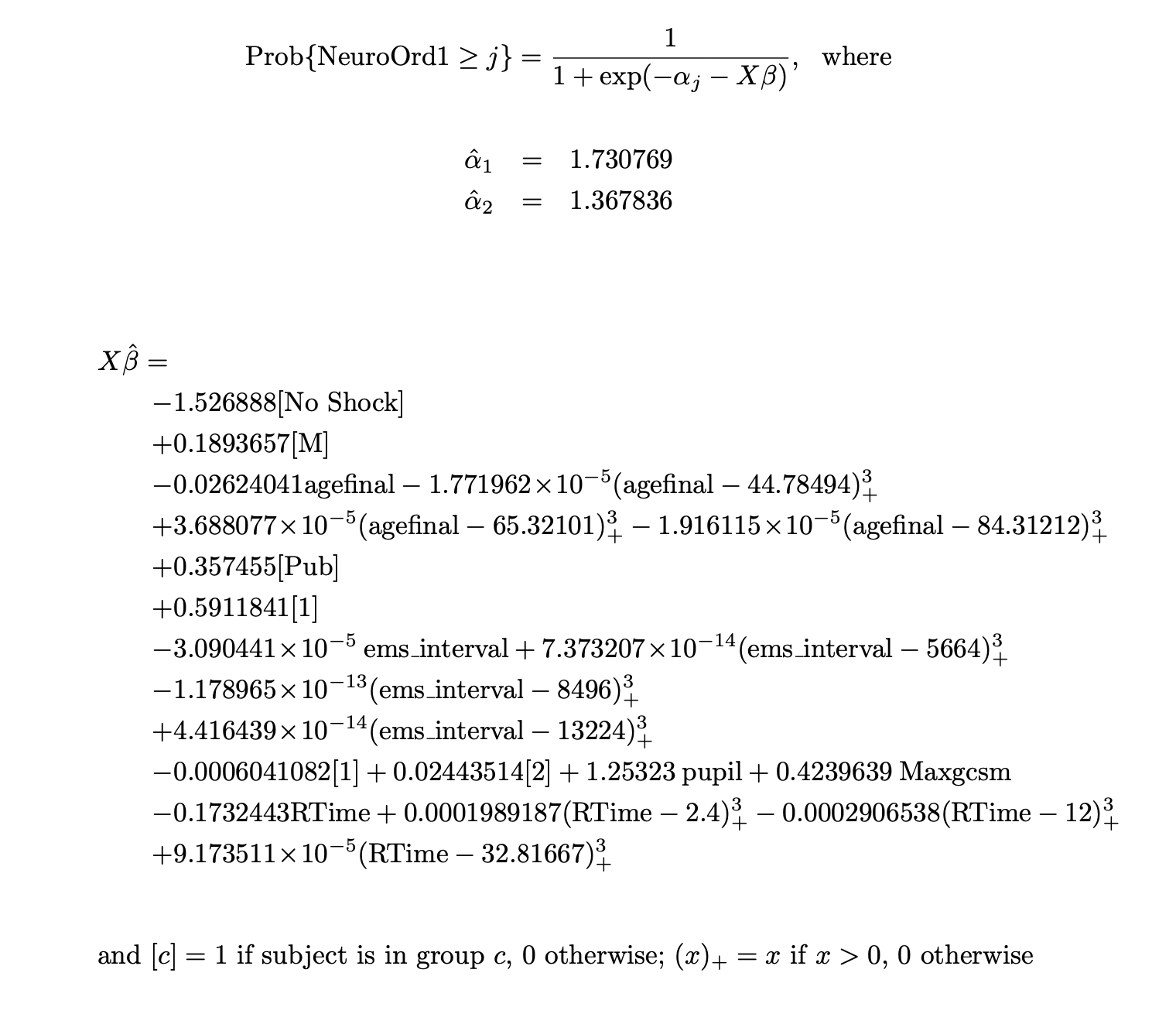

Supplement: Supplementary data 1 [file mmc1.docx]
